# Factors Contributing to Rationed Nursing Care in the Slovak Republic—A Secondary Analysis of Quantitative Data

**DOI:** 10.3390/ijerph19020702

**Published:** 2022-01-08

**Authors:** Dominika Kalánková, Daniela Bartoníčková, Ewelina Kolarczyk, Katarína Žiaková, Agnieszka Młynarska

**Affiliations:** 1 Department of Nursing, Jessenius Faculty of Medicine in Martin, Comenius University in Bratislava, 036 01 Martin, Slovakia; bartonickovadaniela@gmail.com (D.B.); katarina.ziakova@uniba.sk (K.Ž.); 2 Department of Nursing, Faculty of Health Sciences, Palacký University in Olomouc, 775 15 Olomouc, Czech Republic; 3 Department of Gerontology and Geriatric Nursing, Faculty of Health Sciences, Medical University of Silesia, 40-055 Katowice, Poland; ekolarczyk@sum.edu.pl (E.K.); amlynarska@sum.edu.pl (A.M.)

**Keywords:** rationed care, secondary analysis, contributory factors, nurses

## Abstract

Rationed nursing care is a significant problem in healthcare facilities worldwide. Awareness of contributing factors to rationed care might support the development and implementation of strategies for reducing this phenomenon from clinical practice. The study examined the association between selected hospital, unit, and staff variables and the prevalence of rationed nursing care. Secondary analysis of cross-sectional data collected between December 2017 and July 2018 from 895 registered nurses in seven acute care hospitals in the Slovak Republic was performed. Data were collected using the questionnaire Perceived Implicit Rationing of Nursing and analyzed by descriptive and inferential statistics in the statistical program SPSS 25.0. Statistically significant associations were found between rationed nursing care and unit type, education, shift type, nurses’ experience in the current unit, overtime hours, missed shifts, intention to leave the position, perceived staff adequacy, quality of patient care, and job satisfaction. Differences in rating rationed nursing care, quality of patient care, and job satisfaction were identified based on hospital type. Together with top hospital management, nurse managers should develop targeted interventions focusing on mitigating rationed nursing care from the clinical practice with a focus placed on university hospitals. Quality and safe care might be ensured through constant monitoring of the quality of patient care and job satisfaction of nurses as these factors significantly predicted the estimates of rationed nursing care.

## 1. Introduction

Rationed nursing care represents a significant topic within the nursing researchers’ community. Especially, in the past twenty years, the number of publications related to the phenomenon of rationed nursing care has increased. In literature, rationed nursing care is defined as withholding necessary nursing care activities by nurses during their working shifts. The phenomenon occurs in the healthcare system when available resources are insufficient to deliver adequate nursing care to patients. These resources refer mainly to labor or material resources, communication, teamwork, or time [1]. Worldwide, up to 98% of registered nurses (RNs) have not provided at least one nursing care activity to patients during their working shifts [2].

Nowadays, more emphasis is placed on the quality of nursing care, patient safety, patient outcomes, teamwork, satisfaction with the work environment, and job satisfaction of nurses. Moreover, cost-effective, safe, competent, and high-quality care is required when providing health care [1,2]. However, the prevalence of rationed nursing care significantly affects patient safety and the quality of nursing care. Hence, it is necessary to map the prevalence of this phenomenon in acute or long-term care healthcare facilities, primary or community settings, but mainly to identify factors that contribute to its prevalence with the aim to plan strategies targeted at the reduction or elimination of rationed nursing care from the nursing practice [2].

Based on the literature review [3], many factors may contribute to the prevalence of rationed nursing care worldwide. One of the most significant factors is the nurse–patient ratio [4,5,6] and the overall number of staff in the care unit [7,8]. An inadequate number of registered nurses significantly predicts the rationed nursing care in various clinical settings [4] and closely relates to withholding necessary activities mainly in the nursing care areas such as patient surveillance, communication, or adequate documentation of care [4]. Shift types were identified as another factor escalating the estimates of rationed nursing care. Nurses working at daily shifts ration more nursing care activities than nurses working at night shifts mainly due to the excessive demands on care during the day, including examinations, laboratory tests, and physicians’ prescriptions [4,9]. Furthermore, more rationed care is presented by nurses working full-time [10,11] or those with lower qualifications [12]. Several authors also pointed out the differences in the prevalence of rationed care based on the unit type [13,14,15,16]. For example, nurses working at medical-surgical care units report more rationed care than those working at intensive care units (ICUs); however, the estimates of rationed nursing care are still high at ICUs [2,8,14]. In several studies [11,17,18], nurse education was significantly associated with rationed nursing care. Nurses with lower education (secondary or higher education/diploma in nursing) report more nursing care activities being rationed compared to nurses with bachelor’s or master’s degrees in nursing. Nurse education is a factor that, however, varies across countries because of inconsistencies in curricula; hence, results of international studies examining this factor against rationed nursing care might differ significantly [4]. In several studies, nurses’ experiences in the current position or overall nursing experiences were determined as other meaningful factors contributing to rationed nursing care [15,17,18]. In several studies [17,18], more experienced nurses reported rationing more nursing care activities. Interestingly, according to Blackman et al. [12], less experienced nurses were more likely to ration treatment-related care. Other important factors contributing to rationed nursing care that was addressed in many studies are nurse age [12,14,18,19,20], overtime hours [7,9], absenteeism [12], or perceived staff adequacy [14,21,22].

Based on a review of evidence on factors contributing to rationed nursing care, we may conclude that these factors are pretty well examined internationally. However, the literature lacks the consensus on what factors should be monitored in different countries on a national level. Authors from different socio-cultural contexts provide different contributory factors, often specifically reporting those related to the healthcare organization [4,7,12]. However, several recent studies pointed out that also the quality of patient care and job satisfaction of nurses should be examined against the rationed nursing care [1,13,14]. Moreover, little is known about contributing factors to rationed nursing care in the Slovak Republic. Several studies focusing on identifying rationed nursing care revealed its high estimates, up to 40% among Slovak nurses [23]. Furthermore, approximately 87.6% of Slovak RNs rationed at least one nursing care activity [24]. Based on high estimates of rationed nursing care among Slovak nurses, examining contributory factors to rationed care seems essential in planning and implementing targeted strategies for its elimination from clinical practice. Therefore, our study aimed to explore which hospital, unit, and staff variables, including quality of patient care and job satisfaction of nurses, contribute to the prevalence of rationed nursing care in selected teaching and university hospitals in the Slovak Republic.

## 2. Materials and Methods

### 2.1. Design

The study had the characteristics of a cross-sectional study based on a secondary analysis of data from the questionnaire Perceived Implicit Rationing of Nursing Care (PIRNCA) completed from December 2017 to July 2018. In the primary study, the main objective was to study the prevalence and patterns of rationed nursing care and analyze the psychometric properties of the questionnaire PIRNCA. The current study used the original data sample to focus on what the survey data might reveal about associations between selected hospital, unit, and staff variables and the prevalence of rationed nursing care.

### 2.2. Participants

The sample included 895 registered nurses from five teaching hospitals and two university-based hospitals in the Slovak Republic. Registered nurses were selected using the purposeful method. Nurses were included if they provided direct patient care to adult patients, worked at standard care units or intensive care units, and were shift-working. In contrast, nurses were not included if they worked at pediatrics, gynecology–obstetrics, or anesthesiology care units and had occupied managerial positions.

An online sample size calculator (Qualtrics^®^) was used in the primary study to determine the adequate sample size. Between 2017 and 2018, approximately 40,000 registered nurses worked in healthcare organizations in the Slovak Republic. We used the confidence interval of 95% and, due to the size of the sample, the study obtains a margin of error of ±5%. The sample size was calculated as 391 respondents. Overall, 1456 questionnaires were distributed, and 896 were returned. One questionnaire was excluded due to its incompletion, giving the final number of 895 questionnaires. The sample characteristics are reported elsewhere [24].

### 2.3. Data Collection 

The institutional ethics committee approved the research study. Data on the prevalence of rationed nursing care was collected using the PIRNCA questionnaire [25]. The instrument was validated in the national study on the sample of Slovak registered nurses [23] and consequently analyzed further using different statistical methods [24]. The PIRNCA consists of 31 items, including fundamental and specific nursing care activities, which relate to several areas, such as assistance with physical care, emotional support and education, patient surveillance, coordination of care and discharge planning, documentation of care, and implementation of the prescribed treatment plan. The instrument contains two additional variables—job satisfaction and quality of patient care [25]. Respondents are supposed to evaluate their ability to carry out necessary nursing care activities during the past seven working shifts on the 5-point frequency scale (0—“not needed”, 1—“never”, 2—“rarely”, 3—“sometimes”, 4—“often”) in the PIRNCA instrument. Job satisfaction and job experience are evaluated using the 10-point Likert scale. The single item of job satisfaction is using the following response options: 0–10 (0—not satisfied, 10—very satisfied). Similarly, the single item of job experience uses the same response options (0–10) with different interpretations (0—dangerously poor quality, 10—high-quality care). The questionnaire also contained several items concerning selected hospital, unit, and staff variables. In line with the study objectives, the following variables were analyzed concerning the prevalence of rationed nursing care: unit type, education, age, shift type, nurses’ experience in the current unit, nurses’ experience in the nursing profession, overtime hours in past three months, missed shifts in past three months, intention to leave the position, perceived staff adequacy, job satisfaction, and quality of patient care. The reliability of the instrument is high, representing the value of 0.95 for the Cronbach alpha coefficient.

### 2.4. Data Analysis

Data analysis was carried out in the statistical program IBM SPSS Statistics 25.0. Descriptive statistics such as mean, frequencies, and standard deviation of the sample and study variables were obtained, including the PIRNCA instrument analysis. The mean differences in the variables (unit type, education, age, shift type, nurses’ experiences in the current unit, nurses’ experiences in the nursing profession, overtime hours in past three months, missed shifts in past three months, intention to leave the position, perceived staff adequacy, quality of patient care, and job satisfaction) according to the prevalence of rationed care were analyzed by one-way ANOVA with post hoc Bonferroni’s test as the data met the assumption for normal distribution. In line with study objectives, multiple regression analysis was used to examine the predictors of rationed nursing care in selected teaching and university hospitals in Slovakia. Further, the Pearson chi-squared test (χ^2^) was used to compare the proportion of rationed nursing care between job experience and job satisfaction. For the purposes of the analysis, the quality of patient care was recoded into three options (poor quality, 0–3 points out of 10; moderate quality, 4–7 points out of 10; excellent quality, 8–10 points out of 10). Moreover, job satisfaction was recoded into three options (low level of satisfaction, 0–3 points out of 10; moderate level of satisfaction, 4–7 points out of 10; high level of satisfaction, 8–10 points out of 10). Results were tested on the significance level *p* ≤ 0.05.

## 3. Results

### 3.1. Distribution of Answers of the PIRNCA Instrument

The mean composite score for the entire PIRNCA instrument was 1.74 (SD = 0.91), which indicated that care tended to be rarely rationed rather than sometimes. The most often rationed nursing care activity was a timely response to request/need in less than 5 min, and the least rationed nursing care activity was changing intravenous access sites, tubing, and/or dressing. Descriptive analysis of rationing of nursing care based on the PIRNCA instrument is reported in Table 1.

### 3.2. Analysis of the Relationship between Estimates of Rationed Nursing Care and Hospital, Staff, and Unit Variables

Our study identified ten significant factors contributing to the prevalence of rationed nursing care in the selected teaching and university hospitals in the Slovak Republic. Statistically significant differences in the prevalence of rationed nursing care based on the nurse age and nurses’ experience in the nursing profession were not confirmed (Table 2). According to ANOVA, there were statistically significant differences between the prevalence of rationed nursing care and unit type (*p =* 0.000). A post hoc test revealed that rationed nursing care was significantly lower at ICUs (*p =* 0.000). Significant differences were also identified between rationed nursing care and education (*p =* 0.001). Nurses who had completed secondary vocational school reported significantly less rationed nursing care (*p =* 0.017). Significant differences were confirmed between rationed nursing care and shift type (*p =* 0.027). A post hoc test revealed that nurses working only at night shifts reported significantly lower estimates of rationed nursing care. Differences were also found between nurses’ experience in the current unit and rationed nursing care (*p =* 0.005). Nurses working at the current position less than five years reported significantly lower estimates of rationed nursing care (*p =* 0.000). Significant differences were also identified between rationed nursing care and overtime hours (*p =* 0.009), with lower rationed nursing care reported by nurses who had no overtime hours (*p =* 0.015). Differences were also confirmed between rationed nursing care and missed hours in the past three months (*p =* 0.025), with lower estimates of rationed nursing care reported by nurses who have missed no hours due to illness or injury (*p =* 0.014). According to ANOVA, there were statistically significant differences between the prevalence of rationed nursing care and the intention to leave the current position (*p =* 0.000). A post hoc test revealed that rationed nursing care was significantly reported as lower by nurses with no plans to leave the position (*p =* 0.000). Significant differences were also verified between rationed nursing care and perceived staff adequacy (*p =* 0.000). Nurses who perceived adequate staffing 100% of the time reported significantly less rationed nursing care.

### 3.3. Regression Analysis of Rationed Nursing Care and Selected Hospital, Staff, and Unit Variables

Multiple regression analysis was conducted to examine the relationship between the prevalence of rationed nursing care and selected potential predictors (socio-demographic characteristics). We assessed the multicollinearity by the variance inflation factor (VIF), which identifies the correlation between independent variables and the correlation strength. The VIF values were between 1.07 and 1.37, considered acceptable and excluded multicollinearity. In regression, Model 1 (R^2^ = 0.284; Adj R^2^ = 0.274; F = 28.64; *p* = 0.000) revealed that independent variables explained 28.4% of the prevalence of rationed nursing care. Rationed nursing care was significantly predicted by the number of overtime hours in the past three months, perceived staff adequacy, quality of patient care, and job satisfaction (Table 3).

Higher estimates of rationed nursing care were reported by nurses with more overtime hours in the past three months (β = 0.090, *p* = 0.019). Moreover, nurses, who perceived staff adequacy more of the time, reported, at the same time, higher prevalence of rationed nursing care (β = 0.094, *p* = 0.008). Furthermore, nurses who subjectively evaluated the quality of patient care higher at their workplace, reported less rationed nursing care in their practice in the past seven working shifts (β = −0.135, *p* = 0.000). Moreover, nurses who were more satisfied in their job reported less rationed nursing care in the past seven working shifts (β = −0.110, *p* = 0.000).

### 3.4. Evaluation of Quality of Patient Care and Job Satisfaction among Nurses and Its Association with the Estimates of Rationed Nursing Care

The average number of points for measuring the quality of patient care was 7.94 (SD = 1.69), which indicates that nurses evaluated the quality of care provided to their patients as high. Similarly, the average number of points assessing the job satisfaction was 7.24 (SD = 2.58), which indicates that nurses were satisfied with their current job.

Quality of patient care was significantly associated with rationed nursing care estimates (*p* = 0.000). Significantly lower rationed nursing care was reported by nurses who evaluated the quality of care at their unit as high (8–10 points out of 10) (*p* = 0.000) than those who evaluated the quality of care as moderate (4–7 points out of 10) (*p* = 0.000) or poor (0–3 points out of 10) (*p* = 0.000). Similarly, significant differences were confirmed between job satisfaction and rationed nursing care (*p* = 0.000). Lower rationed nursing care was reported by nurses who were very satisfied with the work on the current unit (8–10 points out of 10) (*p* = 0.000) than those who were moderately satisfied (4–7 points out of 10) (*p* = 0.000) or not satisfied (0–3 points out of 10) (*p* = 0.000).

Further, we evaluated differences in rating rationed nursing care, quality of patient care, and job satisfaction based on hospital type (university or teaching hospital). Results are reported in Table 4. Nurses who worked at university hospitals evaluated the significantly worse quality of patient care than those working in teaching hospitals. Similarly, nurses who worked at university hospitals reported a significantly lower level of satisfaction than those working at teaching hospitals. Moreover, nurses working at university hospitals reported significantly higher estimates of rationed nursing care.

## 4. Discussion

Our secondary analysis examined factors contributing to the prevalence of rationed nursing care in selected teaching and university hospitals in the Slovak Republic. This is the first study investigating contributing factors to rationed nursing care in our setting. Previous studies conducted in our setting revealed a high prevalence of rationed nursing care, up to 40% [23,24]; however, in European countries, the estimates of rationed care were lower than 30% [8]. The higher prevalence of rationed nursing care in selected teaching and university hospitals indicates the phenomenon’s severity and topicality in Slovakia. It might be associated with the socio-cultural context, healthcare system, and nurse shortage. Until 2018, tens of thousands of RNs were missing in the healthcare system in Slovakia, and the number of RNs was continuously decreasing [23]. In line with international studies [2,8,13,14], up to 89.0% of RNs rationed one or more nursing care activities necessary for the patients in the past seven working shifts. However, the percentage of RNs who ration one or more nursing care activities might even reach 100% internationally [2]. Therefore, knowledge about factors leading to the higher estimates of rationed nursing care allows the developing and implementing of appropriate interventions to reduce the negative impact of rationing on patient and nurse outcomes.

Overall, we identified ten significant factors related to the hospital, unit, and staff variables. A significant factor contributing to the rationed nursing care was unit type. Several authors pointed out differences in the rationed nursing care across unit types [13,14,18]. Worldwide, a higher prevalence of rationed nursing care was identified at medical-surgical care units, which was also confirmed in our study [11,13,17]. Lower estimates of rationed care at ICUs might be explained through specific work organization at ICUs, a different method of nursing care provision, higher nurse-to-patient ratio, nurses´ technical skills, and the necessity to monitor patients every hour [26]. Another important factor contributing to rationed care was nurse education. The results of international studies have a contradictory character and only partially support our findings. Several studies indicated that nurses with lower education (such as secondary vocational school) reported more rationed nursing care than those with university-based education in nursing [18,19,20]. However, we identified that nurses with lower education in nursing reported less rationed nursing care, which is supported by two studies [17,27]. This variability might be explained through differences in knowledge and awareness of antecedents of rationed nursing care [9]. Nurses with university education might be confronted with the phenomenon of rationed nursing care during their studies. Moreover, respondents in our study might limit their responses in the context of social desirability, which is the main limitation of self-reported measures [28]. Our study also determined shift type as a significant factor of rationed nursing care. Higher estimates of rationed care were reported by nurses working daily shifts. In the Slovak Republic, most nurses work in daily 12 h shifts, during which the workload is the most prominent (basic nursing care, treatment plan, examinations, administration and application of pharmacotherapy, documentation of care, etc.). Night shifts include mainly the administration of morning pharmacotherapy, monitoring, and patient surveillance. Based on this, a higher prevalence of rationed nursing care will be presented on daily shifts [4,9,15]. Nurses’ experiences in the current unit were identified as another significant factor of rationed nursing care. Higgs et al. [18] stated that with increasing experiences, estimates of rationed nursing care increase as well. However, we found out that the highest prevalence of rationed care was reported by nurses who worked between 11 to 15 years, and the least prevalence was reported by those who worked less than five years at the current position. Nurses whose experiences were less than five years might be considered novice nurses who were full of expectations, enthusiasm, had a caring attitude, and tried to meet all patients’ needs while integrating into the nursing team and the new practice environment. On the contrary, those nurses might be afraid of expressing their ability (inability) to provide all necessary nursing care activities in writing because of potential consequences, such as violations in working relationships, decreased incentive payment, or verbal cautions from nurse managers [29]. Interestingly, nurses’ experiences in the nursing profession were not a significant factor contributing to rationed care in our study. For example, Ausserhofer et al. [10] and Bacaksiz et al. [9] stated that nurses more experienced in the nursing profession declared less rationed nursing care. In contrast, several studies contradicted these results and identified that less experienced nurses reported a higher prevalence of rationed care [12,19,21]. In any case, more attention should be placed on this factor, as the results vary across different socio-cultural contexts. Overtime hours in the past three months were significantly associated with the prevalence of rationed nursing care. Similar results were reported in various countries [9,17]. The statistics related to the number of overtime hours of nurses working in the Slovak Republic has shown that nurses had 3.4% of overtime hours out of the overall number of overtime hours. The number of overtime hours closely relates to the inadequate staffing of the particular care units. Alarmingly, the nurse shortage might be deepened since the highest number of working nurses is between 40 to 50 years old [30]. Naturally, with nurse shortages, the number of overtime hours increases. However, overtime hours have been confirmed to be a significant factor contributing to adverse events and errors [31]. This problem applies to the Slovak Republic and dominates in the international context as well. Moreover, missed shifts in the past three months due to illness or injury were significantly associated with the prevalence of rationed nursing care. Nurses who did not miss any shift reported less rationed nursing care, which is supported by the study of Kalisch et al. [15]. However, no other study has been found in order to support our results. The number of missed shifts might be caused by various factors, such as exhaustion or fatigue, headache, or joint pain [32]. These factors might be related to nurses’ physical and mental workload in particular care units, which is also conditioned by inadequate staffing. Another contributing factor was the intention to leave the position. Nurses with no plans to leave the position reported less rationed nursing care, which is supported by several international studies [33,34,35]. Many factors might influence the intention to leave the position, such as job satisfaction, organizational commitment, quality of work-life, work environment, teamwork, leadership, and the number of overtime hours [36,37]. The results of international studies have shown that the simultaneous effect of several factors violates the professional identity of nurses, which facilitates their decisions in the context of intention to leave the position and thus forces them to withhold nursing care activities for patients [38]. These factors could negatively affect nurses working in teaching and university hospitals in the Slovak Republic and potentiate their intention to leave the position. Perceived staff adequacy was determined to be a significant factor contributing to the prevalence of rationed nursing care. Nurses who perceived adequate staffing 100% of the time reported less rationed nursing care. In contrast, nurses who perceived adequate staffing 0% of the time reported significantly higher estimates of rationed care. This trend was confirmed in many international studies [9,13,14]. Perceived staff adequacy reflects the global problem of the nurse shortage and appears to be a significant issue in selected teaching and university hospitals in the Slovak Republic.

Nurses’ quality of patient care and job satisfaction are two major factors that significantly influence nursing care performance [4,13]. Both factors significantly predicted rationed nursing care in selected hospitals in the Slovak Republic. Regarding the quality of patient care, our results verified the findings of several international studies [4,13,34,39], which point to the fact that the better the nurse subjectively evaluates the quality of care, the less rationed nursing care is reported. At the same time, if nurses evaluate the quality of care as average to dangerously low, they report more rationed nursing care [1,4]. Quality of nursing care, even if only in terms of its subjective evaluation, significantly affects rationed nursing care [40]. Similarly to the quality of patient care, our results describing job satisfaction align with many international studies [13,39,41], which confirmed that the more satisfied nurses were in their work, the less rationed care was reported. Job satisfaction positively affects the overall work performance of nurses [33], and hence the nurses’ ability to ensure all necessary nursing care activities to patients.

Our study revealed differences in rating rationed nursing care, quality of patient care, and job satisfaction based on hospital type. Nurses who worked at university hospitals reported significantly worse quality of patient care, lower job satisfaction, and significantly higher estimates of rationed nursing care than those working in teaching hospitals. These differences might be explained by the greater number of beds, providing more specialized but also different types of procedures and examinations, leading to greater patient care demands. Furthermore, university hospitals dispose of many medical and nursing students during daily shifts [23]. In line with several studies [4,14], the workload of nurses working in university hospitals is higher than those working in teaching hospitals, which are also smaller in terms of the number of beds and have a smaller number of students during the daily shifts of nurses.

The study has several limitations. The first limitation might be the selection method of respondents (purposive method) and the research design (cross-sectional study), so our results cannot be generalized to the whole population of nurses. In contrast, the validity of our results is supported by the sample size. Several previous studies have indicated the risk factors for rationed nursing care, so the study’s usefulness might be limited to the specific socio-cultural context. On the contrary, the study used an adequate methodological design congruent with the study objectives, which supports the strengths of our study. Another limitation is the utilization of self-reported questionnaires in the context of social desirability. The last limitation is evaluating clinically relevant variables (quality of patient care, job satisfaction) by only one item.

## 5. Conclusions

The phenomenon of rationed nursing care significantly jeopardizes the quality of nursing care and patient safety, especially in teaching and university hospitals. Rationed nursing care should be evaluated regularly, especially on care units with a demonstrably higher prevalence of this phenomenon. Hospital management should regularly assess factors contributing to rationed nursing care and address them to eliminate them from the clinical practice. Except for primarily organizational factors such as human resources, overtime hours or missed shifts, job satisfaction and quality of patient care should be acknowledged by hospital management as these significantly predicted rationed nursing care, especially in university hospitals. Therefore, quality of patient care and job satisfaction should be the priority for managing nursing teams to effectively provide quality and safe nursing care and prevent the prevalence of rationed nursing care. Based on the results of our study, these factors should be constantly monitored by nurse managers in university hospitals in the Slovak Republic. Our study might act as an example of examining factors contributing to the prevalence of rationed nursing for further research studies.

## Figures and Tables

**Table 1:** Rationing of nursing care based on PIRNCA questionnaire.

Items *	*N*	Never Rationing (*n* (%))	Rarely Rationing (*n* (%))	Sometimes Rationing (*n* (%))	often Rationing (*n* (%))
1. Routine hygiene care	894	629 (70.3%)	169 (18.9%)	68 (7.6%)	28 (3.1%)
2. Routine skin care	894	629 (70.3%)	182 (20.4%)	72 (8.1%)	11 (1.2%)
3. Changing soiled bed linen	894	490 (54.8%)	250 (28.0%)	121 (13.5%)	33 (3.7%)
4. Assistance with needed ambulation	894	504 (56.4%)	271 (30.3%)	94 (10.5%)	25 (2.8%)
5. Mobilization of changing patient position	894	497 (55.6%)	270 (30.2%)	102 (11.4%)	25 (2.8%)
6. Timely assistance with bowel or bladder elimination	894	523 (58.5%)	251 (28.1%)	100 (11.2%)	20 (2.2%)
7. Assistance with the intake of food or fluid	894	580 (64.9%)	201 (22.5%)	97 (10.8%)	16 (1.8%)
8. Promotion of physical comfort	894	554 (62.0%)	239 (26.8%)	82 (9.1%)	19 (2.1%)
9. Administer medications	894	700 (78.3%)	138 (15.4%)	48 (5.3%)	8 (1.0%)
10. Administer enteral or parenteral nutrition	894	717 (80.2%)	128 (14.3%)	41 (4.6%)	8 (1.0%)
11. Provide wound care	892	663 (74.4%)	182 (20.4%)	37 (4.1%)	10 (1.1%)
12. Change intravenous access sites, tubing, and/or dressing	893	668 (74.8%)	172 (19.2%)	47 (5.3%)	6 (0.7%)
13. Adherence to recommended guidelines for safe patient handling	894	463 (51.8%)	247 (27.6%)	114 (12.8%)	70 (7.8%)
14. Adhere to infection control practices	893	602 (67.4%)	211 (23.6%)	58 (6.5%)	12 (2.5%)
15. Providing the amount of teaching for the patient or his/her family	894	440 (49.2%)	308 (34.5%)	105 (11.7%)	41 (4.6%)
16. Preparing patients for treatments, tests, or procedures	894	618 (69.2%)	207 (23.1%)	58 (6.5%)	11 (1.2%)
17. Emotional or psychological support	894	383 (42.8%)	284 (31.8%)	171 (19.1%)	56 (6.3%)
18. Monitoring of the patient’s physiological status	894	624 (69.8%)	180 (20.1%)	78 (8.7%)	12 (2.1%)
19. Monitoring of the patient’s affect and behavior	894	406 (45.5%)	293 (32.8%)	154 (17.2%)	40 (4.5%)
20. Monitoring of the patient’s physical safety	894	507 (56.7%)	240 (26.8%)	123 (13.8%)	24 (2.7%)
21. Following up on patient status changes	894	451 (50.4%)	313 (35.0%)	104 (11.6%)	26 (3.0%)
22. Timely response to request/need in less than 5 min	894	285 (31.9%)	324 (36.2%)	210 (23.5%)	75 (8.4%)
23. Important conversation with team members	894	361 (40.4%)	309 (34.6%)	182 (20.4%)	42 (4.6%)
24. Important conversation with an external agency	894	497 (55.6%)	227 (25.4%)	134 (15.0%)	36 (4.0%)
25. Important conversation with a patient or family member about discharge	895	486 (54.3%)	253 (28.3%)	128 (14.3%)	28 (3.1%)
26. Provide adequate supervision of or follow-up on delegated activities	895	442 (49.4%)	293 (33.1%)	130 (14.5%)	26 (3.0%)
27. Reviewing the multidisciplinary patient documentation	894	408 (45.6%)	297 (33.2%)	151 (16.9%)	38 (4.3%)
28. Documentation of the initiation or revision of plan of care	893	501 (56.1%)	269 (30.1%)	95 (10.6%)	28 (3.2%)
29. Documentation of assessments and monitoring activities	895	487 (54.4%)	275 (30.7%)	101 (11.3%)	31 (3.6%)
30. Documentation of all of the nursing care provided	893	472 (52.9%)	271 (30.3%)	103 (11.5%)	47 (5.3%)
31. Evaluation of the plan of care	895	455 (50.8%)	293 (32.7%)	103 (11.5%)	43 (5.0%)

* Abbreviated items of the PIRNCA instrument (the instrument cannot be used or reproduced without the written permission of Dr T. Jones).

**Table 2:** Hospital, unit, and staff variables, and overall score (M) and standard deviations (SD) (scale range 0–4) for rationed nursing care (*N* = 895).

Variables	(*n* (%))	M ± SD	Test Statistics	Post Hoc Test
Unit type			13.596 *	
1. Surgical	278 (31.1%)	1.43 ± 0.64		
2. Medical	281 (31.3%)	1.53 ± 0.63		
3. Elderly care	28 (3.1%)	2.13 ± 0.54		1 > 4 *
4. ICU	279 (31.2%)	1.32 ± 0.58		2 > 4 *
5. Other	29 (3.3%)	0.97 ± 0.39		3 > 4 *
Education				
1. Secondary education	221 (24.7%)	1.35 ± 0.65		
2. Higher education	242 (27.1%)	1.49 ± 0.63		
3. Bachelor’s degree	200 22.2%)	1.53 ± 0.60	1.343 *	3 > 1 *
4. Master’s degree or higher	232 (26.0%)	1.57 ± 0.61		4 > 1 *
Age			1.014	
1. 20–30	128 (14.3%)	1.49 ± 0.55		
2. 31–40	208 (23.2%)	1.53 ± 0.66		
3. 41–50	363 (40.6%)	1.49 ± 0.60		
4. 51–60	184 (20.6%)	1.51 ± 0.66		
5. More than 60	12 (1.3%)	1.15 ± 0.53		
Shift type				
1. Daily shifts	254 (28.4%)	1.51 ± 0.68		1 > 2 *
2. Night shifts	38 (4.2%)	1.43 ± 0.68	1.289 *	
3. Rotates	603 (67.4%)	1.48 ± 0.60		3 > 2 *
Nurses’ experience in the current unit				
1. Up to 5 years	257 (28.7%)	1.43 ± 0.61		
2. 6–10 years	183 (20.5%)	1.59 ± 0.60		
3. 11–15 years	103 (11.5%)	1.64 ± 0.55	3.750 *	3 > 1 *
4. 16–20 years	99 (11.1%)	1.55 ± 0.63		
5. More than 21 years	253 (28.2%)	1.43 ± 0.64		
Nurses´ experience in the nursing				
profession				
1. Up to 5 years	128 (14.3%)	1.36 ± 0.58	1.023	
2. 6–10 years	86 (9.6%)	1.41 ± 0.63		
3. 11–15 years	86 (9.6%)	1.58 ± 0.74		
4. 16–20 years	118 (13.2%)	1.40 ± 0.60		
5. More than 21 years	477 (53.3%)	1.12 ± 0.48		
Overtime hours				
1. None	254 (28.4%)	1.39 ± 0.63		
2. Less than 12 h	225 (25.1%)	1.48 ± 0.60	4.718 *	
3. More than 12 h	416 (45.5%)	1.54 ± 0.63		3 > 1 *
Missed shifts				
1. None	614 (68.6%)	1.49 ± 0.60		
2. 1 shift	124 (13.8%)	1.43 ± 0.64		
3. 2–3 shifts	86 (9.6%)	1.37 ± 0.71	3.313 *	
4. More than 4 shifts	71 (8.0%)	1.66 ± 0.69		4 > 1 *
Intention to leave the position				
1. In the next 6 months	43 (4.8%)	1.68 ± 0.65		
2. In the next years	110 (12.3%)	1.79 ± 0.80	1.281 *	1 > 3 *
3. No plans to leave	742 (82.9%)	1.43 ± 0.58		2 > 3 *
Perceived staff adequacy				
1. 100% of the time	73 (8.2%)	1.37 ± 0.51	1.720 *	
2. 75 % of the time	309 (34.6%)	1.38 ± 0.56		
3. 50% of the time	301 (33.6%)	1.50 ± 0.59		
4. 25% of the time	166 (18.5%)	1.66 ± 0.67		4 > 1 *
5. 0% of the time	46 (5.1%)	1.88 ± 0.96		5 > 1 *

* *p* ≤ 0.05; > Bonferroni´s post hoc analysis referring to significant differences in groups.

**Table 3:** Predictors of rationed nursing care among Slovak nurses.

Variables	Rationed Nursing Care	
	β **	*p*
(Constant)		0.000 *
Unit type	−0.001	0.980
Age	0.087	0.373
Education	0.060	0.152
Shift type	−0.085	0.277
Nurse experience in the nursing profession	0.052	0.566
Nurse experience in the current position	−0.002	0.958
Overtime hours	0.090	0.019 *
Missed hours	−0.005	0.885
Intention to leave the position	−0.049	0.167
Perceived staff adequacy	0.094	0.008 *
Quality of patient care	−0.135	0.000 *
Job satisfaction	−0.110	0.000 *

* *p* ≤ 0.05; ** standardized beta coefficient.

**Table 4:** Comparison of rationed nursing care across the hospital type.

Variable	Kinds	University Hospitals	Teaching Hospitals	Pearson’s (df) χ^2^ Test
Quality of patient care	Poor quality	93 (35.6%)	156 (24.6%)	χ^2^ (3) = 171.312 *p* ≤ 0.05
	Moderate quality	145 (55.5%)	329 (51.9%)	
	Excellent quality	23 (8.9%)	149 (23.5%)	
Job satisfaction	Low level of satisfaction	104 (39.8%)	225 (35.4%)	χ^2^ (3) = 97.886 *p* ≤ 0.05
	Moderate level of satisfaction	132 (50.7%)	348 (54.9%)	
	High level of satisfaction	25 (9.5%)	61 (9.6%)	
Rationed nursing care	% of rationing less than never	113 (43.3%)	454 (71.6%)	χ^2^ (2) = 154.792 *p* ≤ 0.05
	% of rationing greater than never	148 (56.7%)	180 (28.4%)	
Total		261 (29.2%)	634 (70.8%)	

## Data Availability

The data presented in the study are available on request from the corresponding author. The data are not publicly available due to ethical and privacy restrictions.
